# Gender Differences in the Mental Health Impact of Natural Disaster Exposure Among Older Adults

**DOI:** 10.1093/geroni/igaf122.3259

**Published:** 2025-12-31

**Authors:** Yeon Jin Choi, Karen Lawrence

**Affiliations:** University of Kentucky, Lexington, Kentucky, United States; University of Kentucky, Lexington, Kentucky, United States

## Abstract

Natural disasters are becoming more frequent and severe, posing significant risks to human health and well-being. The U.S. is among the most disaster-prone countries, with over 85 million people affected by natural disasters in 2016 alone. Prior research links disaster exposure to poor mental health outcomes, yet less is known about how these effects differ by gender, particularly among older adults. Women are generally more vulnerable to mental health disorders following stressful or traumatic experiences, yet the extent to which these gender differences manifest in disaster-related mental health outcomes remains unclear. We examined whether the association between natural disaster exposure and depressive symptoms and anxiety varies by gender, using data from the 2010/2012 Health and Retirement Study (N = 13,419; Mage=65.12, SD = 10.3; female: 54.5%). Among the study sample, 19% of men and 17% of women reported natural disaster exposure. A series of weighted ordinary least squares regression models, stratified by gender, were estimated, adjusting for sociodemographic characteristics. Results indicated that natural disaster exposure was associated with increased depressive and anxiety symptom severity in both men and women. However, associated symptom severity differed by gender: men reported higher depressive symptom severity scores (β=.22, p<.01) than women (β=.14, p<.05), whereas women reported higher anxiety symptom severity scores (β=.08, p<.01) than men (β=.09, p<.001). These findings highlight the differential mental health impact of natural disasters by gender. Disaster response efforts should consider gender-specific vulnerabilities and provide tailored mental health resources to address the unique needs of older men and women.

